# Use of The Global Alliance for Musculoskeletal Health survey module for estimating the population prevalence of musculoskeletal pain: findings from the Solomon Islands

**DOI:** 10.1186/s12891-018-2198-0

**Published:** 2018-08-16

**Authors:** D. G. Hoy, T. Raikoti, E. Smith, A. Tuzakana, T. Gill, K. Matikarai, J. Tako, A. Jorari, F. Blyth, A. Pitaboe, R. Buchbinder, I. Kalauma, P. Brooks, C. Lepers, A. Woolf, A. Briggs, L. March

**Affiliations:** 1 University of Sydney, Sydney, Australia; 2 Global Alliance for Musculoskeletal Health, Truro, UK; 3 Pacific Community (SPC), Noumea, New Caledonia; 4 Solomon Islands National Statistics Office, Honiara, Solomon Islands; 5 University of Adelaide, Adelaide, Australia; 6 Monash University, Melbourne, Australia; 7 University of Melbourne, Melbourne, Australia; 8 Royal Cornwall Hospital, Truro, UK; 9 Curtin University, Perth, Australia

**Keywords:** Musculoskeletal, Pain, Survey, Global, Solomon Islands

## Abstract

**Background:**

Musculoskeletal (MSK) conditions are common and the biggest global cause of physical disability. The objective of the current study was to estimate the population prevalence of MSK-related pain using a standardized global MSK survey module for the first time.

**Methods:**

A MSK survey module was constructed by the Global Alliance for Musculoskeletal Health Surveillance Taskforce and the Global Burden of Disease MSK Expert Group. The MSK module was included in the 2015 Solomon Islands Demographic and Health Survey. The sampling design was a two-stage stratified, nationally representative sample of households.

**Results:**

A total of 9214 participants aged 15–49 years were included in the analysis. The age-standardized four-week prevalence of activity-limiting low back pain, neck pain, and hip and/or knee pain was 16.8, 8.9, and 10.8%, respectively. Prevalence tended to increase with age, and be higher in those with lower levels of education.

**Conclusions:**

Prevalence of activity-limited pain was high in all measured MSK sites. This indicates an important public health issue for the Solomon Islands that needs to be addressed. Efforts should be underpinned by integration with strategies for other non-communicable diseases, aging, disability, and rehabilitation, and with other sectors such as social services, education, industry, and agriculture. Primary prevention strategies and strategies aimed at self-management are likely to have the greatest and most cost-effective impact.

**Electronic supplementary material:**

The online version of this article (10.1186/s12891-018-2198-0) contains supplementary material, which is available to authorized users.

## Background

Musculoskeletal (MSK) conditions are common and the biggest global cause of physical disability [1]. A series of systematic reviews on the prevalence of MSK pain that were conducted as part of the Global Burden of Disease (GBD) 2010 Study [2–8] revealed a substantial shortage of data at the population level in most countries. Further, of the population-level studies available, there was substantial heterogeneity between the case definitions used, making it difficult to compare data across countries and over time.

Many other health conditions causing a large global burden of disease have standard surveys and/or survey modules for estimating population-wide prevalence. For example, for mental health, there is the World Mental Health Survey [9], and the WHO STEPs mental health module [10]. In the absence of a standard survey module for MSK health, characterized by the experience of MSK-related pain, the Global Alliance for MSK Health (GMUSC) embarked on a project to develop such a tool.

The Solomon Islands is a nation that sits in the Pacific region. Like many areas of the world, there is very limited information on MSK pain in the Pacific [11]. The objective of the current study was to use the GMUSC MSK survey module to estimate the prevalence of pain at common MSK sites in the Solomon Islands.

## Methods

### Study setting

The Solomon Islands is a low-income country and is part of the Melanesian group of islands. It consists of nearly 1000 islands and has a tropical climate. English is the official language, but Pidgin is also widely used. Most of the country’s labor force is engaged in subsistence crop and animal raising, hunting and fishing. In 2016, the population was estimated to be approximately 640,000 with approximately 80% living in rural areas. In 2009, life expectancy at birth was estimated to be 66 years for males and 73 years for females. While infectious diseases continue to be prominent in the Solomon Islands, non-communicable diseases (NCDs) are also rapidly increasing due largely to changing lifestyles [12].

### Study design

The GMUSC MSK survey module was included in the 2015 Solomon Islands Demographic and Health Survey (SIDHS). The Global Demographic and Health Survey (DHS) Program has conducted the DHS more than 300 times in over 90 countries. It selects nationally-representative samples to study fertility, family planning, maternal and child health, gender, HIV/AIDS, malaria, and nutrition [13]. The SIDHS focused on these issues as well as pain of MSK aetiology (referred to as ‘MSK pain’).

The SIDHS was coordinated by the Solomon Islands National Statistics Office (SINSO) with assistance from the Pacific Community (SPC). Training for conducting the survey was conducted at three levels according to standard DHS training procedures: 1) Training of trainers; 2) Pilot training; and 3) Main training. Training of trainers included training of those who would lead subsequent trainings, as well as testing of the questionnaires for content, translation, skip procedures and filtering instructions. The pilot training of fieldworkers was provided to 70 field workers from five provinces and was also another opportunity to further test the questionnaires. The field workers consisted of SINSO staff, nurses, health technicians, and trained interviewers. All the field workers could read, speak and understand Pidgen, and most, but not all, were literate in English. The main training took 3 weeks and included 145 field workers. It included presentations, mock interviews, quizzes and role-playing. Data were collected by 14 teams, each of nine people. Data collection initially ran from April to September 2015, and a follow-up period ran from October to November 2015 to revisit those people who were not available to respond to the survey at their initial visit. This was limited to the capital, Honiara, as this was the site where the majority of non-responders lived.

### Participants

The sampling design was a two-stage stratified, nationally-representative sample of households. In the Solomon Islands, enumeration areas (EAs) are at the sub-village level and are considered by the SINSO to be the smallest geographical unit. The average size of an EA is 68 households. There are a total of 1342 EAs and 91,251 households in the Solomon Islands [12]. A target sample size of 5064 households was chosen by the SINSO for consistency with the previous SIDHS in 2006/7, and took into account an anticipated household response rate of 95%.

The sampling frame was a list of all EAs and their respective number of households. From this list, 211 EAs were selected using sampling with probability proportional to the estimated number of households in the EA. In each of the selected EAs, 24 households were selected using systematic random sampling. Consistent with standard DHS protocol, which has a focus on women’s reproductive health, all women aged 15–49 in these households were eligible to be individually interviewed, while men aged 15 and over in every second household were eligible to be interviewed. For this reason, women aged 50 years and over were not sampled.

### Variables, data sources and measurement

Three questionnaires (household, female, and male) were used [12]. All content except for the MSK module was based on previously developed modules by the Global DHS Program, which were adapted to the specific needs of the Solomon Islands.

The MSK survey module was constructed by the GMUSC Surveillance Taskforce and members of the GBD MSK Expert Group. The intention was to develop a one-page module that would not necessarily be used as a stand-alone tool, but rather as a component that could seamlessly integrate within other pre-existing and planned surveys such as national health surveys, or in this case, the DHS. Such purposive integration would likely increase its uptake, save resources, minimise survey burden from conducting multiple surveys on local communities, provide access to a larger range of covariates, and encourage responses that are integrated with other NCDs and other health issues such as healthy aging, and disability, rather than having ‘siloed’ MSK responses.

The MSK module was based on literature reviews and previous consensus exercises for establishing a standard approach to collecting population-based data on the epidemiology of various MSK conditions. The measures in the MSK module were prevalence of low back pain, neck pain, and hip and/or knee pain. For low back pain, we used the minimal definition of low back pain for face-to-face interviews from an international modified Delphi consensus study [14]. For neck pain, the results from a previous expert consensus exercise were used [15]. For hip and/or knee pain, the results from an expert consensus exercise for monitoring MSK problems in Europe were used [16]. Consistent with the methods of a previous systematic review of prevalence studies of low back pain [17], four key elements were specified in the module: the prevalence period (4 weeks), the anatomical location referred to; a minimum episode duration (1 day), and whether or not the question referred to activity-limiting pain. For hip and/or knee pain, it was felt a question on diagnosis for people with chronic symptoms was also important to include. It was felt this was less important for spinal pain given, for example, the importance of avoiding over-medicalization of back pain [18]. The module is shown in Additional file 1.

Using standard DHS protocols, the survey questionnaires were translated into Pidgin and back-translated into English in order to check the accuracy of the translation. Survey content, skip procedures and filtering instructions were pre-tested by staff from the SINSO.

### Efforts to minimize study bias

To minimise risk of bias in the prevalence estimates of MSK pain we used a framework based upon a risk of bias tool for prevalence studies [19]. Specifically: 1) The MSK module items had been developed through extensive Delphi and other expert consensus exercises; 2) The case definitions used were in accordance with international recommendations; 3) Selection bias was minimized through selecting a nationally-representative sample using standard selection methods; 4) Non-response bias was minimized by conducting follow up visits to non-responders; 5) Data were collected directly from the subjects as opposed to from a proxy; 6) The same mode of data collection (face-to-face interviews) was used for all subjects; and 7) The recall period was 4 weeks – this length results in less recall bias than longer periods (e.g., 1 year).

### Statistical methods

Data were entered into the CSPro computer package two times with 100% verification [20]. Analysis was performed in Microsoft Excel [21]. For the purpose of comparison between the genders, we only included males aged 15 to 49 years for most of the analysis. The outcome variables of interest were activity-limiting low back pain (AL-LBP), activity-limiting neck pain (AL-NP), activity-limiting hip or knee pain (AL-HKP), and hip or knee pain that had lasted for longer than 3 months (HKP > 3/12). There were relatively little missing data: 16 missing values (out of 9214) for AL-LBP, 14 for AL-NP, 4 for AL-HKP, and 39 for HKP > 3/12. These were treated conservatively as a ‘No’ response in the analysis.

Proportions and confidence intervals (CIs) for each outcome variable of interest were calculated. An overall design effect (averaged over all variables) had been calculated for the SIDHS to account for the complex sampling design [12] – this value (1.393) was applied in the calculation of CIs. The age-standardized prevalence for the age-group 15–49 years for each outcome variable of interest was calculated using Solomon Islands national population data for 2015. Sub-group analyses were conducted for gender, age group, urbanicity (urban households were considered to be those in Honiara or provincial capital towns), maximum education level reached, and ethnicity. We performed further analysis on prevalence in males across all ages 15 years and above to assess whether prevalence estimates were likely to be under or over estimates for the population 15 years and above in the Solomon Islands.

## Results

### Study participants

**Fig. 1:**
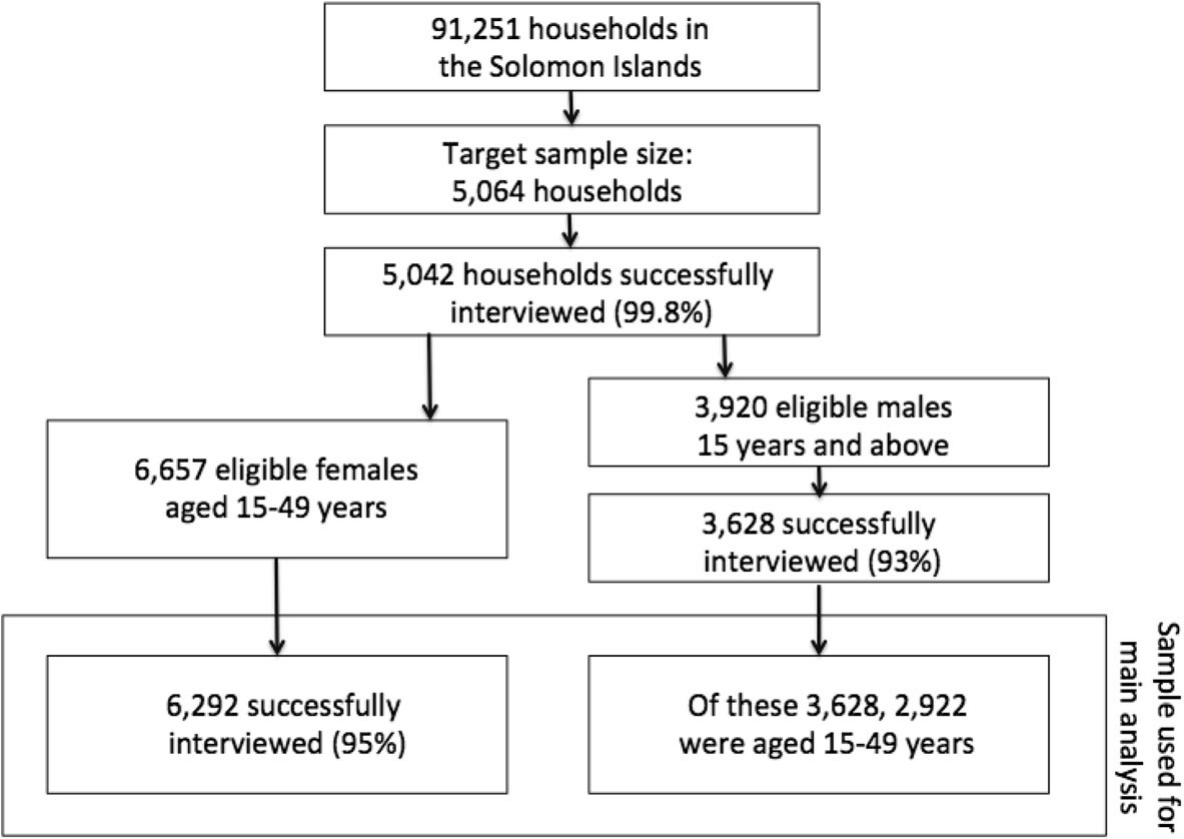
Flowchart of sample selection

Of the 5064 households selected in the sample, 5042 households (99.8%) (*n* = 9214 participants) were successfully interviewed. Among the households interviewed, 6657 eligible females 15–49 years were identified, of whom 6292 (95.0%) were successfully interviewed. For males, 3920 eligible males were identified, of whom 3628 (93.0%) were successfully interviewed. Of these, 2922 (80.6%) were aged 15–49 years (Fig. 1).

Of the interviewees, 5429 (59%) resided in rural areas, 3750 (41%) were educated to pre-school or primary level, 4032 (44%) to secondary level, and 721 (8%) to tertiary level. 8857 (96%) were Melanesian, 202 (2.2%) Polynesian, 131 (1.4%) Micronesian, and the remainder from some other ethnic group.

### Activity-limiting low back pain

The four-week prevalence of low back pain was 50.5% (95% CI: 49.3 to 51.7). The four-week prevalence of activity-limiting low back pain was 16.8%; the four-week age-standardized prevalence of activity-limiting low back pain was 16.7% (95% CI: 16.4 to 16.9). Prevalence was significantly higher in females compared with males, and rural areas compared with urban areas. Across both sexes, prevalence generally tended to increase with age and decrease by education level. Prevalence was highest in Micronesians, followed by Melanesians, and then Polynesians (Table 1).

**Table 1:** Four-week prevalence of activity-limiting low back pain (“In the past 4 weeks, have you had pain in your low back? If yes, was this pain bad enough to limit your usual activities or change your daily routine for more than one day?”), both sexes, 15–49 year olds

Background characteristic	Numerator	Denominator	Prevalence (%)	LCL (95%)	UCL (95%)
Total	1543	9214	16.8	15.9	17.7
Sex					
Female	1166	6292	18.5	17.4	19.7
Male	377	2922	12.9	11.5	14.3
Age group					
15–19	182	1818	10.0	8.4	11.6
20–24	234	1582	14.8	12.7	16.9
25–29	240	1534	15.7	13.5	17.8
30–34	249	1341	18.6	16.1	21.0
35–39	240	1204	19.9	17.3	22.6
40–44	201	959	21.0	17.9	24.0
45–49	197	776	25.4	21.8	29.0
Urbanicity					
Urban	532	3785	14.1	12.8	15.4
Rural	1011	5429	18.6	17.4	19.8
Education					
Pre-school	2	9	22.2	0.0	54.3
Primary	723	3741	19.3	17.8	20.8
Secondary	597	4032	14.8	13.5	16.1
Tertiary	81	721	11.2	8.5	14.0
Vocational	25	148	16.9	9.8	24.0
Other	1	156	0.6	0.0	2.1
Missing	114	555	20.5	16.6	24.5
Ethnicity					
Melanesian	1488	8857	16.8	15.9	17.7
Polynesian	30	202	14.9	9.1	20.6
Micronesian	24	131	18.3	10.5	26.1
Other	1	21	4.8	0.0	15.5
Missing	0	3	0.0	0.0	0.0

*LCL* Lower 95% Confidence Limit, *UCL* Upper 95% Confidence Limit

### Activity-limiting neck pain

The four-week prevalence of neck pain was 29.6% (95% CI: 28.5 to 30.7). The four-week prevalence of activity-limiting neck pain was 8.92%; the four-week age-standardized prevalence of activity-limiting neck pain was 8.9% (95% CI: 8.7 to 9.1). Prevalence was higher in females compared with males, although this was not significant at the 0.05 level. Prevalence was significantly higher in rural areas compared with urban areas. Again, prevalence generally tended to increase with age and decrease by education level across both sexes. Prevalence was highest in Melanesians, followed by Micronesians, and then Polynesians (Table 2).

**Table 2:** Four-week prevalence of activity-limiting neck pain (“In the past 4 weeks, have you had pain in your neck? If yes, was this pain bad enough to limit your usual activities or change your daily routine for more than one day?”), both sexes, 15–49 year olds

Background characteristic	Numerator	Denominator	Prevalence (%)	LCL (95%)	UCL (95%)
Total	822	9214	8.9	8.2	9.6
Sex					
Female	596	6292	9.5	8.6	10.3
Male	226	2922	7.7	6.6	8.9
Age group					
15–19	123	1818	6.8	5.4	8.1
20–24	134	1582	8.5	6.9	10.1
25–29	116	1534	7.6	6.0	9.1
30–34	118	1341	8.8	7.0	10.6
35–39	129	1204	10.7	8.7	12.8
40–44	101	959	10.5	8.2	12.8
45–49	101	776	13.0	10.2	15.8
Urbanicity					
Urban	287	3785	7.6	6.6	8.6
Rural	535	5429	9.9	8.9	10.8
Education					
Pre-school	2	9	22.2	0.0	54.3
Primary	391	3741	10.5	9.3	11.6
Secondary	320	4032	7.9	7.0	8.9
Tertiary	43	721	6.0	3.9	8.0
Vocational	15	148	10.1	4.4	15.9
Other	1	156	0.6	0.0	2.1
Missing	50	555	9.0	6.2	11.8
Ethnicity					
Melanesian	804	8857	9.1	8.4	9.8
Polynesian	10	202	5.0	1.4	8.5
Micronesian	7	131	5.3	0.8	9.9
Other	1	21	4.8	0.0	15.5
Missing	0	3	0.0	0.0	0.0

*LCL* Lower 95% Confidence Limit, *UCL* Upper 95% Confidence Limit

### Activity-limiting hip or knee pain

The four-week prevalence of hip or knee pain was 33.9% (95% CI: 32.8 to 35.1). The four-week prevalence of activity-limiting hip or knee pain was 10.8%; the four-week age-standardized prevalence of activity-limiting hip or knee pain was 10.8% (95% CI: 10.5 to 11.0). Prevalence was significantly higher in females compared with males, and rural areas compared with urban areas. Across both sexes, prevalence increased with age, and tended to decrease by education level. Prevalence was highest in Melanesians, followed by Micronesians, and then Polynesians (Table 3).

**Table 3:** Four-week prevalence of activity-limiting hip or knee pain (“In the past 4 weeks, have you had pain in your hips or knees? If yes, was this pain bad enough to limit your usual activities or change your daily routine for more than one day?”), both sexes, 15–49 year olds

Background characteristic	Numerator	Denominator	Prevalence (%)	LCL (95%)	UCL (95%)
Total	998	9214	10.8	10.1	11.6
Sex					
Female	779	6292	12.4	11.4	13.3
Male	219	2922	7.5	6.4	8.6
Age group					
15–19	116	1818	6.4	5.1	7.7
20–24	142	1582	9.0	7.3	10.6
25–29	142	1534	9.3	7.6	11.0
30–34	150	1341	11.2	9.2	13.2
35–39	164	1204	13.6	11.3	15.9
40–44	129	959	13.5	10.9	16.0
45–49	155	776	20.0	16.7	23.3
Urbanicity					
Urban	328	3785	8.7	7.6	9.7
Rural	670	5429	12.3	11.3	13.4
Education					
Pre-school	1	9	11.1	0.0	35.3
Primary	486	3741	13.0	11.7	14.3
Secondary	365	4032	9.1	8.0	10.1
Tertiary	48	721	6.7	4.5	8.8
Vocational	15	148	10.1	4.4	15.9
Other	1	156	0.6	0.0	2.1
Missing	82	555	14.8	11.3	18.3
Ethnicity					
Melanesian	971	8857	11.0	10.2	11.7
Polynesian	13	202	6.4	2.4	10.4
Micronesian	14	131	10.7	4.4	16.9
Other	0	21	0.0	0.0	0.0
Missing	0	3	0.0	0.0	0.0

*LCL* Lower 95% Confidence Limit, *UCL* Upper 95% Confidence Limit

### Hip or knee pain lasting longer than three months

The data collectors accidentally asked about *any* hip or knee pain that had lasted for 3 months or more, as opposed to *activity-limiting* hip or knee pain that had lasted for 3 months or more. The four-week prevalence of any hip or knee pain that had lasted for 3 months or more was 10.6% (95% CI: 9.9 to 11.4); the four-week age-standardized prevalence of any hip or knee pain that had lasted for 3 months or more was 9.9% (95% CI: 9.7 to 10.1). Prevalence was significantly higher in females (11.8%; 95% CI: 10.9 to 12.7) compared with males (8.0%; 95% CI: 6.9 to 9.2), and rural areas (12.5%; 95% CI: 11.5 to 13.6) compared with urban areas (7.9%; 95% CI: 6.9 to 8.9). Prevalence increased with age in both sexes, and decreased by education level in males; there was no clear relationship between prevalence and education level in females. Prevalence was highest in Melanesians, followed by Micronesians, and then Polynesians.

### Diagnosis given to those who had hip or knee pain over the past month

Similar to the previous question, the data collectors accidentally asked for the past-month diagnosis related to *any* hip or knee pain that had lasted for 3 months or more, as opposed to the diagnosis related to *activity-limiting* hip or knee pain that had lasted for 3 months or more. Of the 3125 people who had experienced hip or knee pain over the past 4 weeks, 18.2% (*n* = 570) reported they had received a diagnosis from a medical doctor. The most common diagnoses given were pneumonia (*n* = 228), common cold (*n* = 161), and bone dislocation (*n* = 14).

### Activity-limiting MSK pain in multiple MSK sites

Of the 22.1% (*n* = 2035) of people who had activity-limiting MSK pain at one or more of the three sites examined, 11.7% (*n* = 1093) had activity-limiting MSK pain at one site only, 6.0% (*n* = 556) at two sites, and 4.2% (*n* = 386) at all three sites.

### Age trends of activity-limiting pain

**Fig. 2:**
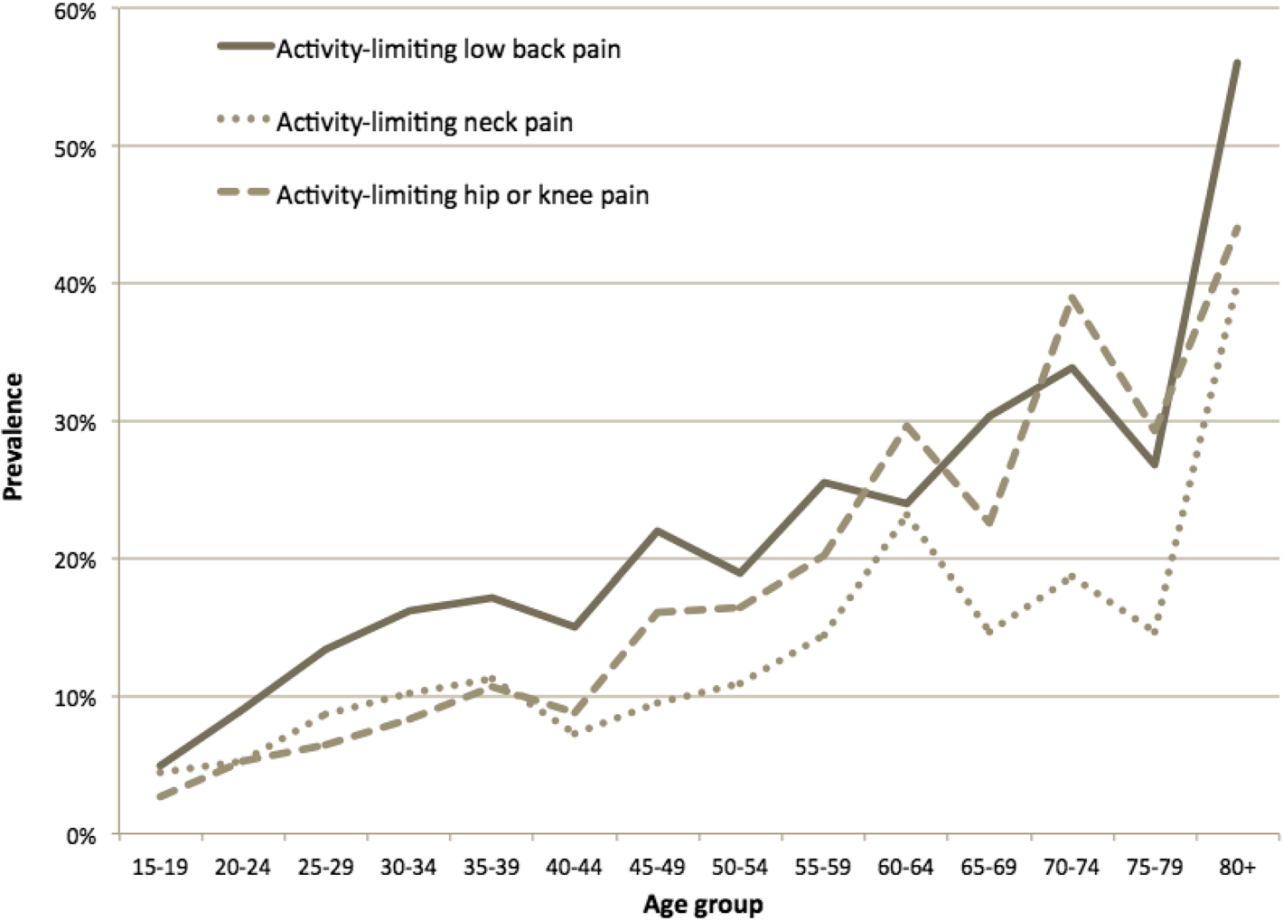
Four-week prevalence of activity-limiting pain, males, 15 years and above

There was a substantial increase in the prevalence of pain at all MSK sites in the years beyond 49 years of age for males, suggesting that the estimates in the 15–49 year age group are likely to be an underestimate of prevalence for all people 15 years and above in the Solomon Islands (Fig. 2).

### Use of the MSK module

The SINSO and SPC staff reported that the GMUSC MSK module integrated well with the DHS survey, was easy to understand and use, and that it only added a few extra minutes to each interview. The use of the MSK module and analysis of MSK data for the Solomon Islands study cost a few thousand dollars compared to the tens or even hundreds of thousands of dollars a stand-alone survey of this size would have cost. Data analysis indicated only 1% of data were missing.

## Discussion

The objectives of the current study were to derive standard and comparable estimates of the prevalence of common MSK pain in the Solomon Islands; and to use the GMUSC MSK module for the first time, and in particular, to determine whether it could feasibly be integrated within an existing population health survey.

### Strengths and limitations

We surveyed a nationally representative sample of 15–49 year olds (and a wider age-range in males), and used a standardized tool, which integrated into an existing national survey instrument. A number of measures were taken to minimize study bias, and there was a very good response rate. The recall period of 4 weeks may have resulted in some recall bias. One minor error by data collectors occurred in that for question MSK 7 and MSK8, they used question MSK 5 as the entry point, rather than question MSK 6, thus including *any* hip or knee pain rather than *activity-limiting* hip or knee pain. This will be made clearer in future trainings.

### MSKs in the Solomon Islands

The prevalence of pain was high at all the MSK sites that were evaluated. Low back pain had the highest prevalence, followed by hip or knee pain, and then neck pain. Our findings for the age-standardized prevalence of activity-limiting low back and neck pain in the Solomon Islands were substantially higher than those of GBD 2016 (low back pain: 5.1%; neck pain: 2.6%), which are based on models that extrapolate data from elsewhere [22]. This is partly, but not entirely, explained by the inclusion of all ages in GBD 2016 estimates. The ratio between the age-standardized prevalence of activity-limiting low back and neck pain (1.87) was comparable to the same ratio for GBD 2016 (1.96) [22].

Consistent with previous research on MSK conditions, prevalence of MSK pain tended to increase with age in each of the sites that we evaluated [17, 23, 24]. The results were also consistent with previous findings that low back pain tends to be more prevalent in those with lower levels of education [25]. One of the most interesting findings was in regard to ethnicity. Generally, Polynesian countries have a higher prevalence of overweight and obesity compared to Melanesian and Micronesian countries [26]. Given this, and the fact that obesity is a risk factor for low back pain [25], and osteoarthritis of the knee [27], one may have expected higher rates of low back and knee pain in the Polynesian group in this study. However, this group had the lowest prevalence, although this was not significant at the 0.05 level. Further research is needed to better understand this.

The diagnoses reported for hip and/or knee pain were most commonly pneumonia and the common cold. There are high rates of communicable diseases in the Solomon Islands and it is possible that these symptoms reflect manifestations of infection [28]. While SPC report there was not an unusually high amount of influenza-like illnesses being reported in the Solomons at the time of the survey there was a dengue fever outbreak and reported cases of Zika virus [29], and both of these can result in arthralgia. [30, 31]. These self-reported diagnoses may also reflect a lack of knowledge about causes of MSK pain among the general population and/or communication gaps with health professionals.

This study has established that MSK pain is common, and given existing data on the burden of disease associated with MSK conditions, a significant burden of disease is likely to be present in the Solomon Islands. This likely current and future burden needs to be better addressed in order to maximise function, productivity and livelihoods, and ensure that health systems in the Solomons are not overwhelmed as the country’s population ages. However, in upscaling efforts to address the burden of MSK in the Solomons, it is important that a vertical or ‘siloed’ approach to addressing MSKs is avoided.

It is key that any approach addressing the burden of MSKs is underpinned by ‘integration’ [32, 33]. MSK pain shares risk factors that are common to other conditions such as diabetes and cardiovascular disease. Further, as people age, they are faced with multi-morbidities and require an integrated approach to health care to ensure the care is person-centred, holistic and efficient [34]. Consequently, an approach that is integrated with other non-communicable diseases, issues such as aging, disability, and rehabilitation, and other sectors such as social services, education, industry, and agriculture is likely to promote a more streamlined, cost-effective approach, avoid doubling of efforts and wasting of resources, and avoid ultimate damage to fragile health systems [32]. Integration should take place at macro- (policy) levels, meso- (health service) levels, and micro- (clinician/patient) levels [33].

Primary prevention strategies and strategies aimed at self-management are likely to have the greatest and most cost-effective impact in reducing the burden of MSK pain in the Solomon Islands. For example, Buchbinder et al. promote the concept of living well with low back pain through focusing on self-management and healthy lifestyles [18]. Where feasible, approaches should be integrated with those of other non-communicable diseases, and issues such as healthy aging, consistent with recommendations from the World Health Organization [34]. Locally relevant mass media campaigns encouraging healthy lifestyles, including obesity-reduction and physical activity, and improving population beliefs about prevention and management of MSK health and persistent pain, are likely to have a positive impact [32, 33]. Models of Care need to be tailored to the specific capacities and needs of the specific setting. For example, it may be possible to have dedicated staff for treating MSK pain at the national level; however, at sub-national levels, this may become less realistic given the limited funding and number of staff in health services. Alternative approaches, such as having NCD generalists, inclusive of MSK health, who provide treatment and advice should be further explored.

### MSK module

Staff reported the tool MSK module integrated well, was easy to use, and it was very affordable compared with the alternative of a stand-alone survey. Utilization of the module as part of existing surveys is therefore likely to be a relatively affordable way of improving data coverage across the globe, and thus improving the understanding of MSK pain, and informing ways of mitigating the growing global burden of MSK pain. While in-depth cognitive interviews and examination of inter-rater reliability of the tool was not possible in the current study due to resource constraints and heavy workloads of the data collection team, this is planned for the future.

The other benefit of including the module within other more general surveys, and consistent with the Principles of Development Effectiveness [35–37], is that it can help to facilitate integration of the MSK response across the broader health system. Translation of survey results into national policy and practice aimed at reducing MSK burden is paramount. A two-page summary of findings and key recommendations was prepared for the Solomon Islands government to assist with this process (Additional file 2).

The Global Alliance for Musculoskeletal Health have already used the results of the pilot to further refine the module for use in other countries throughout the world. Questions on upper limb pain have been added, and the tool has recently been piloted in a South Australian health survey. The latest version of the tool can be found at http://bjdonline.org/msk-survey-module/.

## Conclusion

The prevalence of activity-limited pain was high in all measured MSK sites in the Solomon Islands. This indicates an important public health issue for the Solomon Islands that needs to be addressed. Efforts should be underpinned by integration with strategies for other non-communicable diseases, aging, disability, and rehabilitation, and with other sectors such as social services, education, industry, and agriculture. Primary prevention strategies and strategies aimed at self-management are likely to have the greatest and most cost-effective impact.

## Additional files

Additional file 1: Global Alliance for Musculoskeletal Health survey module: This is the survey module that is referred to in the manuscript. (PDF 462 kb)

Additional file 2: Two-page summary of findings and key recommendations for the Solomon Islands government: This is a summary of findings and key recommendations for the Solomon Islands government. (PDF 440 kb)

## Abbreviations

AL-HKP: Activity-limiting hip or knee pain
AL-LBP: Activity-limiting low back pain
AL-NP: Activity-limiting neck pain
CI: Confidence interval
DHS: Demographic and Health Survey
EA: Enumeration Area
GBD: Global Burden of Disease
GMUSC: Global Alliance for Musculoskeletal Health
HIV/AIDs: Human immunodeficiency virus infection and acquired immune deficiency syndrome
HKP > 3/12: Hip or knee pain that had lasted for longer than 3 months
MSK: Musculoskeletal
NCDs: Non-communicable diseases
NHMRC: National Health and Medical Research Council
SIDHS: Solomon Islands Demographic and Health Survey
SINSO: Solomon Islands National Statistics Office
SPC: Pacific Community
UNICEF: United Nations Childrens’ Fund
WHO STEPs: The World Health Organization stepwise approach to surveillance of non-communicable disease risk
