# Supplementary material for: Use of The Global Alliance for Musculoskeletal Health survey module for estimating the population prevalence of musculoskeletal pain: findings from the Solomon Islands
Source: BMC Musculoskelet Disord. 2018 Aug 16;19:292. doi: 10.1186/s12891-018-2198-0 (PMC6097436; doi:10.1186/s12891-018-2198-0)
Supplement: Supplementary file 1 — Global Alliance for Musculoskeletal Health survey module: This is the survey module that is referred to in the manuscript. (PDF 462 kb) [file 12891_2018_2198_MOESM1_ESM.pdf]

Additional file 1: Global Alliance for Musculoskeletal Health survey module

	Question	Diagram	Response	Code
LOW BACK PAIN	In the past 4 weeks, have you had pain in your low back (in the area shown on the diagram)?	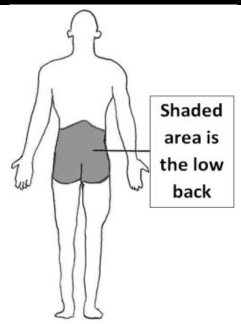 	Yes 1 → go to MSK2<br>No 2 → go to MSK3<br>Refused 88 → go to MSK3	**MSK1**
	If yes, was this pain bad enough to limit your usual activities or change your daily routine for more than one day?		Yes 1 → go to MSK3<br>No 2 → go to MSK3<br>Refused 88 → go to MSK3	**MSK2**
NECK PAIN	In the past 4 weeks, have you had pain in your neck (in the area shown on the diagram)?	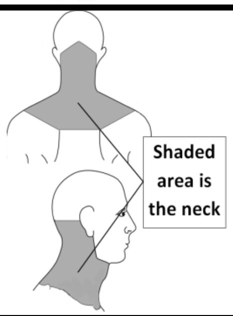 	Yes 1 → go to MSK4<br>No 2 → go to MSK5<br>Refused 88 → go to MSK5	**MSK3**
	If yes, was this pain bad enough to limit your usual activities or change your daily routine for more than one day?		Yes 1 → go to MSK5<br>No 2 → go to MSK5<br>Refused 88 → go to MSK5	**MSK4**
HIP AND KNEE PAIN	In the past 4 weeks, have you had any pain in your hips **or** knees (in the area shown on the diagram)?	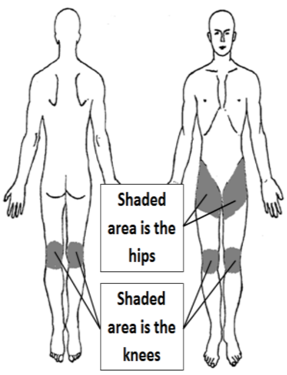 	Yes 1 → go to MSK6<br>No 2 → finish<br>Refused 88 → finish	**MSK5**
	If yes, was this pain bad enough to limit your usual activities or change your daily routine for more than one day?		Yes 1 → go to MSK7<br>No 2 → finish<br>Refused 88 → finish	**MSK6**
	Has this problem lasted for 3 months or more?		Yes 1 → go to MSK8<br>No 2 → finish<br>Refused 88 → finish	**MSK7**
	For this problem, have you been told by a medical doctor what the diagnosis is?		Yes 1 → go to MSK9<br>No 2 → finish<br>Refused 88 → finish	**MSK8**
	If yes, what was the diagnosis?		--------------------------------	**MSK9**

Instructions for interviewer:

- For question MSK1, ask the interviewee the question and show them the diagram of the low back and explain that the low back is defined as the area shaded (which is from the bottom rib to the bottom of the bottom just before it connects with the leg). If they answer yes, go to question MSK2. If they answer no or refuse to answer (to MSK1) go to question MSK3.
- For question MSK3, ask the interviewee the question and show them the diagram of the neck and explain that the neck is defined as the area shaded. If they answer yes, go to question MSK4. If they answer no or refuse to answer (to MSK3) go to question MSK5.
- For question MSK5, ask the interviewee the question and show them the diagram of the hip and knees and explain that they are defined as the area shaded. Note, even one joint (e.g. right hip) qualifies as a 'yes' for this question. If they answer yes, go on to questions MSK6. If they answer no or refuse (to MSK5), this completes the module.
- For question MSK6, ask the interviewee the question. If they answer yes, go on to questions MSK7. If they answer no or refuse (to MSK6), this completes the module.
- For question MSK7, ask the interviewee the question. If they answer yes, go on to question MSK8. If they answer no or refuse (to MSK7), this completes the module.
- For question MSK8, ask the interviewee the question. If they answer yes, go on to question MSK9. If they answer no or refuse (to MSK8), this completes the module.
- For question MSK9, ask the interviewee the question, and wait for their response. Do not give them any suggestions. If they cannot remember the diagnosis, enter 'cannot remember'. Once they answer, this completes the module.
