# Supplementary material for: Use of The Global Alliance for Musculoskeletal Health survey module for estimating the population prevalence of musculoskeletal pain: findings from the Solomon Islands
Source: BMC Musculoskelet Disord. 2018 Aug 16;19:292. doi: 10.1186/s12891-018-2198-0 (PMC6097436; doi:10.1186/s12891-018-2198-0)
Supplement: Supplementary file 2 — Two-page summary of findings and key recommendations for the Solomon Islands government: This is a summary of findings and key recommendations for the Solomon Islands government. (PDF 440 kb) [file 12891_2018_2198_MOESM2_ESM.pdf]

Additional file 2: Two-page summary of findings and key recommendations for the Solomon Islands government

What was done?

- Musculoskeletal (MSK) pain is common and the biggest global cause of physical disability. Like many areas of the world, there is very limited information on MSK pain in the Pacific.

- The objective of the current study was to use a standardized global MSK survey module for the first time, and estimate the prevalence of common MSK pain in the Solomon Islands.

- The one-page MSK module was included in the 2015 Solomon Islands Demographic and Health Survey (SIDHS). The module had been constructed by the Global Musculoskeletal Alliance (GMUSC) Surveillance Taskforce.

What was found?

A total of 9,214 participants aged 15-49 years were included in the analysis. The main findings were as follows:

- Over the past four weeks:
  - One in every six people interviewed (16.8 % of people) had experienced low back pain that had impacted on their ability to do their usual activities or changed their routine for more than one day.
  - One in every nine people interviewed (10.8 % of people) had experienced hip and/or knee pain that had impacted on their ability to do their usual activities or changed their routine for more than one day.
  - One in every eleven people interviewed (8.9 % of people) had experienced neck pain that had impacted on their ability to do their usual activities or changed their routine for more than one day.
- Low back, neck, and hip/knee pain tended to be more common as people got older. It was also more common in those with the lowest education levels.

What is recommended?

- MSK pain creates a substantial burden in the Solomon Islands.
- It is important that efforts to address this burden are upscaled to improve function, productivity and livelihoods, and to ensure that health systems in the Solomons are not overwhelmed as the country's population ages.
- Efforts should ensure that a vertical or 'siloed' approach to addressing MSKs is avoided.
- Instead, it is important that efforts involve integration with efforts to address other non-communicable diseases, and issues such as aging, disability, and rehabilitation.
- Further, integration with other sectors such as education, industry, and agriculture is important.
- Integration should take place at macro- (policy) levels, meso- (health service) levels, and micro- (clinician/patient) levels.

- Primary prevention strategies and strategies aimed at self-management are likely to have the greatest and most cost-effective impact. For example, mass media campaigns encouraging healthy lifestyles, including obesity-reduction and physical activity.
- In regards to treatment of MSK pain, it may be possible to have dedicated staff for treating MSK pain at the national level; however, at sub-national levels, this may become less realistic given the limited funding and number of staff in health departments. Alternative approaches, such as having NCD generalists, inclusive of MSKs, that provide treatment and advice for all MCDs, including MSKs, should be further explored.
- It is important that treatment does not create a reliance on the health-care system. A key focus of treatment should be advice about prevention and self-management.
